# Are Total and Domain-Specific Sedentary Time Associated with Overweight in Older Taiwanese Adults?

**DOI:** 10.3390/ijerph121012697

**Published:** 2015-10-12

**Authors:** Ming-Chun Hsueh, Yung Liao, Shao-Hsi Chang

**Affiliations:** 1 Department of Physical Education, National Taiwan Normal University, No. 162, Heping East Road Section 1, Taipei 10643, Taiwan; E-Mail: boxeo19912016@gmail.com; 2 Department of Health Promotion and Health Education, National Taiwan Normal University, Taipei 10643, Taiwan; E-Mail: liaoyung@ntnu.edu.tw

**Keywords:** sedentary behavior, domain-specific sedentary time, sitting, TV viewing, overweight, obesity, older adults, Taiwan

## Abstract

This study investigated the associations between total and domain-specific sedentary time with the risk of overweight in older adults. A cross-sectional study was conducted by administering computer-assisted telephone interviews to 1046 Taiwanese older adults (aged ≥65 years) residing in two regions in Taiwan in 2015. Odds ratios (ORs) and 95% confidence intervals (CIs) were calculated to examine the associations between self-reported total and six domain-specific sedentary times and body mass index status (calculating by self-reported height and weight) by using logistic regression analyses. The results showed that compared with older women in the lowest quartile of the total sedentary time, those in the highest quartile were 1.87 (95% CI: 1.10–3.21) times more likely to be overweight, after adjustment for potential confounders. The total sedentary time was stratified into six specific domains, and only watching television more than 2 h per day was positively associated with overweight (OR, 1.55; 95% CI: 1.08–2.25) in older women, whereas no other sedentary time domains were associated with the risk of overweight. No significant associations were observed in older men. Further studies using prospective designs are required to confirm the presently observed effects of total and domain-specific sedentary behavior on the health of older adults.

## 1. Introduction

Being overweight is linked to mortality worldwide and constitutes a major risk factor for adverse health outcomes [1]. As in other countries worldwide [2], the prevalence of overweight is increasing among older Taiwanese adults, of whom nearly half (49.1%) are overweight [3]. Thus, developing effective strategies for overweight prevention among Taiwanese older adults is crucial.

Sedentary behavior is defined as any waking behavior that involves a basal metabolic rate ranging from 1.0 to 1.5 metabolic equivalents (METs) in a sitting or reclining posture [4]. A systematic review regarding sedentary behavior and health outcomes indicated that longer total sitting and television (TV) viewing times are respectively associated with higher overweight risk in older adults, whereas time spent sitting in a car is not associated with overweight [5]. However, previous studies have been limited to concurrently comparing the associations of both total and domain-specific sedentary time with the risk of overweight in older adults, even though examining domain-specific sedentary behavior (e.g., computer use, reading, socializing, and transportation) has been emphasized for developing tailored interventions [6]. Moreover, men and women may have different patterns of sedentary behavior [7]. Few studies have examined gender differences in the associations between sedentary behavior and overweight risk in older adults, especially in Asian countries. One study conducted in Spain reported a positive association between overall sitting time and overweight in older women, but not in older men [8]. Therefore, the aim of this study was to examine the associations of total and six domain-specific sedentary times with overweight in Taiwanese older men and women.

## 2. Methods

### 2.1. Respondents

A survey of people aged 65 years or older was conducted using a computer-assisted telephone interviewing system in Taipei City and Chiayi County, Taiwan, in May 2015. Sampling was performed using random-digit dialing. The potential target population comprised 468,922 older adults. A total of 1714 older adults were contacted, and 1095 of them completed the survey (response rate: 63.9%). No incentive was offered to the respondents. The study protocol was approved by the Ethics Committee of National Taiwan Normal University (201504HM004).

### 2.2. Measures

#### 2.2.1. Outcome Variables

Body mass index (BMI) is a valid indicator of health outcomes in older adults [9]. The outcome variable was body mass index (BMI), which was calculated using self-reported height and weight, and dichotomized into non-overweight (<24 kg/m^2^) and overweight (including obesity, ≥24 kg/m^2^) by using Taiwanese cutoff points for older adults [10].

#### 2.2.2. Exposure Variables

A validated exposure scale requiring 1-week recall was used to determine the domain-specific sedentary time [11]. Participants reported on the activities that they performed during the preceding week while they were sitting or lying down (not including time spent in bed) and the total time spent for each activity. The activities were watching TV, videos, or DVDs; using a computer; reading; socializing with friends or family; traveling in a motor vehicle or on public transport; and engaging in hobbies. Taiwanese older adults exhibited an acceptable test-retest reliability (Spearman correlation coefficient = 0.63). The total sedentary times were divided into several quartiles (cut-off points: 2.54, 4.14, 6.5 h/day), and each of the following domains was dichotomized using a median value: TV viewing (high ≥ 2, low < 2 h/day), computer use (=0, >0 h/day), reading (=0, >0 h/day), socializing (≥0.5, <0.5 h/day), transport (≥0.29, <0.29 h/day), and hobbies (=0, >0 h/day).

#### 2.2.3. Covariates

The covariates were age, gender, marital status, job status, educational level, area of residence, and living status (Table 1). Time spent in leisure-time physical activity (LTPA) was included as a confounder for analyses. LTPA was assessed using a part of the International Physical Activity Questionnaire-long version (IPAQ-LV) [12]. IPAQ-LV contains the domains of work, transport, domestic and garden, and leisure time. Only LTPA was used as a covariate in the present study because older adults were observed to spend more time on LTPA than on other activities in the domain of physical activity [13]. Moreover, LTPA was reported to be more closely related to health benefits than other domains in older adults [13]. The total leisure time spent in vigorous-intensity LTPA, moderate-intensity LTPA, and walking was calculated and dichotomized into “sufficient PA” (≥150 min/week) and “insufficient PA” (<150 min/week) according to public health guidelines [14].

### 2.3. Analyses

Following data cleaning, data from 1046 respondents were obtained for analysis. A chi-squared test was conducted to identify proportional differences in the sample characteristics between older men and older women (Table 1). A chi-squared test was conducted to identify proportional differences in the sample characteristics between older men and women (Table 1). Furthermore, a Mann-Whitney U test was executed to calculate the differences in the M (SD) of the total sedentary time and domain-specific sedentary behavior according to gender because the distribution of sedentary behavior was skewed. A binary logistic regression was conducted to estimate the odds ratios (OR) and 95% confidence intervals (CI) for the association of total and domain-specific sedentary times with overweight in the total sample, and separately for older men and older women (Table 2). For total sedentary time, covariates were adjusted for the logistic regression model. For six domain-specific sedentary times, both covariates and other domain-specific sedentary variables were entered simultaneously into the logistic regression model. Analyses were conducted using SPSS Version 24.0 with the level of significance set at *p* < 0.05.

## 3. Results

### 3.1. Characteristics of Participants

Table 1 shows the basic characteristics of the total sample, containing older men and older women. The chi-squared test showed that older women were more likely to be unmarried, unemployed, have a lower educational level, live with family, and have sufficient LTPA. Furthermore, a Mann-Whitney U test was used to determine the differences in total and domain-specific sedentary times between older men and older women. Older men were observed to report a higher total sedentary time, time spent on computer use, socializing, and transport compared with older women. In addition, older women were more likely to report higher TV viewing and reading time compared with older men.

**Table 1:** Sample characteristics stratified by gender, expressed as % or M (SD).

Variable	Category	Total Sample n = 1046 (%)	Older Men n = 491 (46.9)	Older Women n = 555 (53.1)	*p* Value ^a^
Age	65–74 years	643 (61.5)	302 (61.5)	341 (61.4)	0.98
	75+ years	403 (38.5)	189 (38.5)	214 (38.6)	
Marital status	Married	791 (75.6)	413 (84.1)	378 (68.1)	<0.001 **
	Unmarried	255 (24.4)	78 (15.9)	177 (31.9)	
Job status	Employment	201 (19.2)	117 (23.8)	84 (15.1)	<0.001 **
	Not employment	845 (80.8)	374 (76.2)	471 (84.9)	
Education level	College or higher	244 (23.3)	163 (33.2)	81 (14.6)	<0.001 **
	≤High school	802 (76.7)	328 (66.8)	474 (85.4)	
Residential area	Metropolitan	534 (51.1)	236 (48.1)	298 (53.7)	0.07
	Nonmetropolitan	512 (48.9)	255 (51.9)	257 (46.3)	
Living status	Alone	137 (13.1)	50 (10.2)	87 (15.7)	0.009 *
	With family	909 (86.9)	441 (89.8)	468 (84.3)	
LTPA (min/week)	Sufficient (≥150)	449 (39.4)	182 (37.1)	267 (48.1)	<0.001 **
	Insufficient (<150)	597 (60.6)	309 (62.9)	288 (51.9)	
BMI (kg/m^2^)	Non-overweight (<24)	608 (58.1)	281 (57.2)	327 (58.9)	0.58
	Overweight (≥24)	438 (41.9)	210 (42.8)	228 (41.1)	
Total sedentary time (h/day)		4.72 (2.89)	5.04 (2.91)	4.43 (2.83)	<0.001 **
TV viewing (h/day)		2.30 (1.87)	2.17 (1.77)	2.42 (1.95)	0.04 *
Computer use (h/day)		0.44 (1.08)	0.54 (1.21)	0.35 (0.95)	<0.001 **
Reading (h/day)		3.73 (6.21)	2.52 (6.82)	3.04 (5.53)	<0.001 **
Socializing (h/day)		0.83 (0.88)	0.91 (0.98)	0.75 (0.79)	0.03 *
Transport (h/day)		0.45 (0.57)	0.57 (0.66)	0.34 (0.45)	<0.001 **
Hobbies (h/day)		0.15 (0.48)	0.20 (0.59)	0.11 (0.35)	0.08

Notes: ^a^ *p* value for proportional differences between older men and older women according to a chi-squared test for categorical variables and Mann-Whitney U test for continuous variables. Abbreviations: LTPA = leisure time physical activity; BMI = body mass index; M (SD) = mean (standard deviation); h/day = hours per day. * *p* < 0.05, ** *p* < 0.001.

### 3.2. Total and Domain-Specific Sedentary Time Associated with Overweight

Table 2 shows ORs for overweight by categories of total and domain-specific sedentary time, adjusting for confounding factors. In the total sample, compared with the lowest quartile, the highest quartile of the total sedentary time was associated with a higher risk of overweight (OR, 1.51). Furthermore, regarding the associations between domain-specific sedentary time and the risk of overweight, the results revealed that older adults who watched TV for prolong time (≥2 h/day) were 1.32 times more likely to be overweight compared with those who watched TV for a shorter time. No significant associations were observed between other domains of sedentary time. The sample was stratified by gender, and significant associations were observed only in older women. Among older women, compared with lowest quartile of total sedentary time, the highest quartile of total sedentary time was associated with a higher risk of overweight (OR, 1.87). The total sedentary time was stratified into six specific domains, and only older women associated with a prolonged TV viewing time (≥2 h/day) demonstrated a higher likelihood of being overweight (OR, 1.55) compared with women associated with a shorter TV viewing time, whereas no other sedentary time domains (computer use, reading, socializing, transport, and hobbies) were observed. Furthermore, regardless of adjustments for LTPA, significant associations of total sedentary time and TV viewing time with overweight were still observed in older women.

**Table 2:** Adjusted odds for overweight by total and domain-specific sedentary time in older adults; binary logistic regression.

Variable	Total Sample		Older Men		Older Women	
	OR (95% CI)	*p* Value	OR (95% CI)	*p* Value	OR (95% CI)	*p* Value
**Total sedentary time ^a^ (h/day)**						
Quartile 1	**1.00 (Ref.)**		1.00 (Ref.)		1.00 (Ref.)	
Quartile 2	1.15 (0.80–1.65)	0.45	0.85 (0.49–1.50)	0.58	1.45 (0.90–2.32)	0.13
Quartile 3	1.13 (0.78–1.64)	0.53	1.15 (0.67–1.98)	0.61	1.07 (0.63–1.81)	0.80
Quartile 4	1.51 (1.03–2.20)	0.03 *	1.24 (0.72–2.13)	0.44	1.87 (1.10–3.21)	0.02 *
**TV viewing (h/day)**						
Low (<2)	1.00 (Ref.)	0.04 *	1.00 (Ref.)	0.56	1.00 (Ref.)	0.02 *
High (≥2)	1.32 (1.02–1.71)		1.12 (0.77–1.62)		1.55 (1.08–2.25)	
**Computer use (h/day)**						
=0	1.00 (Ref.)	0.79	1.00 (Ref.)	0.61	1.00 (Ref.)	0.99
>0	0.96 (0.70–1.31)		0.89 (0.57–1.38)		1.00 (0.62–1.60)	
**Reading (h/day)**						
=0	1.00 (Ref.)	0.24	1.00 (Ref.)	0.74	1.00 (Ref.)	0.20
>0	0.84 (0.63–1.12)		0.93 (0.61–1.41)		0.77 (0.51–1.16)	
**Socializing (h/day)**						
Low (<0.5)	1.00 (Ref.)	0.28	1.00 (Ref.)	0.22	1.00 (Ref.)	0.60
High (≥0.5)	1.16 (0.89–1.52)		1.29 (0.86–1.93)		1.10 (0.77–1.58)	
**Transport (h/day)**						
<0.29	1.00 (Ref.)	0.63	1.00 (Ref.)	0.67	1.00 (Ref.)	0.31
≥0.29	0.94 (0.71–1.22)		1.09 (0.73–1.64)		0.82 (0.56–1.20)	
**Hobbies (h/day)**						
=0	1.00 (Ref.)	0.24	1.00 (Ref.)	0.96	1.00 (Ref.)	0.09
>0	0.81 (0.57–1.15)		0.99 (0.61–1.61)		0.63 (0.37–1.07)	

Notes: Adjusted for gender, age, marital status, job status, educational level, residential area, living status, and LTPA. Abbreviations: h/day = hours per day; TV = television. ^a^ Quartiles of total sedentary time cut-off point: 2.54, 4.14, 6.5 h/day. * *p* < 0.05.

## 4. Discussion

The study was the first to examine total and 6 domain-specific sedentary times and the risk of overweight concurrently in Taiwanese older adults. The main finding of the present study is that total sedentary time and extensive TV viewing time (≥2 h/day) are concurrently associated with a greater risk of overweight in older women, regardless of LTPA. Our findings are consistent with those of previous studies that reported independent associations of total sitting time with overweight in older women populations [8,15]. Possible reasons for the association of total sedentary and TV viewing times with overweight risk are that TV viewing behavior is a marker of an overall pattern of sedentary behavior in women [7]. Moreover, in the present study, TV viewing time constituted a substantial proportion of the overall sedentary time (48.8%), which is consistent with the finding of a previous study on older adults [16]. In addition, the role of prolonged sedentary time has been recognized as low-energy-expenditure behavior, which burns few calories and leads to weight gain [17]. Furthermore, the main reason for the positive associations between TV viewing and overweight risk may be that TV viewing behavior entails little break time and relatively low energy expenditure compared with other domain-specific sedentary behaviors, such as driving a car (2.0 METs), which may have greater health impact on older adults than other populations [18]. Moreover, prolonged TV viewing was observed to be associated with high energy intake and frequent snacking [19]. These findings may suggest that both total and domain-specific sedentary behavior should be considered for developing effective strategies for overweight prevention in older women.

The results of this study revealed that gender differences in older adults influenced the associations of total and domain-specific sedentary time with overweight. Significant associations of total sedentary and TV viewing times with overweight were observed only in older women. This finding is similar to that of a study conducted in Spain, which reported that sitting time was independently associated with overweight only in older women [8]. A possible explanation is that men and women exhibit dissimilar sedentary lifestyle patterns [7]; particularly, older women might spend more time viewing TV than older men do [20]. In addition, most of older women live at home, fulfilling their traditional role as a housewife or caregiver, and they are unlikely to spend substantial time outside of their home [21]. Therefore, they might spend more leisure time watching TV or exhibit more total sedentary time at home compared with men. Thus, TV viewing may be a crucial factor in reducing sedentary behavior, particularly for older women [7].

Our findings reveal that except for TV viewing, no significant associations were observed between domain-specific sedentary behavior (computer use, reading, socializing, transport, and hobbies) and overweight in older adults. A possible reason is that the energy expenditure in these sedentary activities might be slightly higher than that in TV viewing [18]. Consistent with the findings of previous studies on older Americans [22], the time spent in public transport or a car was not associated with the overweight risk in older adults. A possible explanation for this is that older adults may spend little time in daily traveling and long times at home [21]. Moreover, our participants expended little time engaging in other domain-specific sedentary behaviors (computer use, reading, socializing, transport, and hobbies), which may not contribute to the risk of overweight in older adults. Further study is required to specifically measure domain-specific sedentary behavior and overweight.

This study has several limitations. First, the study had a cross-sectional design; therefore, it was not possible to make causal inferences. Second, the self-reported data used in this study could have led to bias. Third, confounding variables such as dietary behaviors and alcohol consumption were not measured. Fourth, participants were limited to two regions in Taiwan; thus, the data are not nationally representative.

## 5. Conclusions

The cross-sectional characteristics of the study findings limit the conclusions that can be drawn from these findings because a causal link cannot be assumed between total and domain-specific sedentary behavior with the risk of overweight. Another limitation was the inclusion of self-report data of sedentary behaviors, weight, and height; moreover, dietary intake was not assessed. Nevertheless, we found evidence for long total sedentary behavior and TV viewing times were significantly associated with a risk of overweight, particular in older women. These results also have important implications for policy makers or intervention designers for develop effective strategies to minimize sedentary behavior for the prevention and management of older adults’ overweight. Future studies should be considered using prospective designs to confirm the currently observed findings of total and domain-specific sedentary behavior with overweight in older adults.
